# Mandibular non-Hodgkin’s lymphoma: two observations of a challenging disease

**DOI:** 10.11604/pamj.2020.37.102.23770

**Published:** 2020-09-29

**Authors:** Yassine Oueslati, Raouaa Belkacem Chebil, Iyadh Abidi, Badreddine Sriha, Habib Khochtali, Lamia Oualha, Nabiha Douki

**Affiliations:** 1 Department of Oral Medicine and Oral Surgery, Sahloul Hospital, Dental Faculty of Monastir, University of Monastir, Monastir, Tunisia,; 2 Laboratory of Oral Health and Maxillofacial Rehabilitation, University of Monastir, Monastir, Tunisia,; 3 Cytology and Pathological Anatomy Department, Farhat Hached Hospital, University of Sousse, Sousse, Tunisia,; 4 Department of Maxillofacial surgery, Sahloul Hospital, University of Sousse, Sousse, Tunisia

**Keywords:** Non-Hodgkin’s lymphoma, diffuse large B-cell lymphoma, Burkitt lymphoma, mouth, diagnosis, biopsy, chemo-immunotherapy

## Abstract

Lymphomas are a heterogeneous group of malignant tumours of the haematopoietic system characterized by an aberrant proliferation of mature lymphoid cells or their precursors and mainly represented by non-Hodgkin´s lymphomas (NHL). The aim of this paper was to report two cases of NHLs with mandibular locations by detailing their different clinical, radiological, and histopathological aspects, as well as the approach followed to diagnose these diseases and to provide patients with the appropriate therapeutic management. The first case is about a 72-year-old female patient who was diagnosed with a large B-cell lymphoma while the second one concerns a 16-year-old male patient who was diagnosed with a Burkitt’s lymphoma. These observations represent the two highly aggressive known NHLs according to the WHO classification. The mandibular locations of these diseases are rare and represent only 0.6% of all the reported cases. It is important to note that only a deep and good quality tumour biopsy can provide a diagnosis of certainty. The reference treatment is medical consisting in the introduction of chemo-immunotherapy. As oral surgeons, we have an important role in the early diagnosis of these malignancies and in the patient’s referral to specialized care in order to get the appropriate treatment.

## Introduction

Lymphomas are a heterogeneous group of malignant tumours of the haematopoietic system characterized by an aberrant proliferation of mature lymphoid cells or their precursors [1]. Lymphomas can be divided into two major entities: Hodgkin´s lymphoma (HL) which usually arises in the lymph nodes, with a predilection for neck and mediastinal nodes, and non-Hodgkin´s lymphoma (NHL) which can occur in extranodal sites in 40% of the cases and it is by far the most frequent [2]. The oral cavity and the digestive tract are the main sites of extranodal NHLs [3]. These diseases are currently classified based on clinical features, morphology, immunophenotyping and molecular genetics. Various schemes have been developed to classify lymphomas over the years. The one which is widely used is the 2008 World Health Organization (WHO) classification, which is based on the principles defined in the Revised European-American Classification of Lymphoid Neoplasms (REAL) [4]. It was updated in 2016 to provide pathologists and haematooncologists with the recent advances in diseases understanding [5]. Over 20 different subtypes of NHL have been classified according to the specific subtype of the lymphoid cells involved. We recognize B-cell lesions which are the most common (About 85-90% of NHL) and T-cell as well as natural killer-cell lesions (T/NK-NHL) which are less common [6]. Despite the recent major therapeutic advances, NHLs remain a challenge for haematooncologists because of their frequency and severity. In this paper, we report two cases of NHL with mandibular location by detailing their different clinical, radiological, and histological aspects, as well as the approach followed to diagnose these diseases and to provide patients with the appropriate therapeutic management.

## Patient and observation

**Figure 1:**
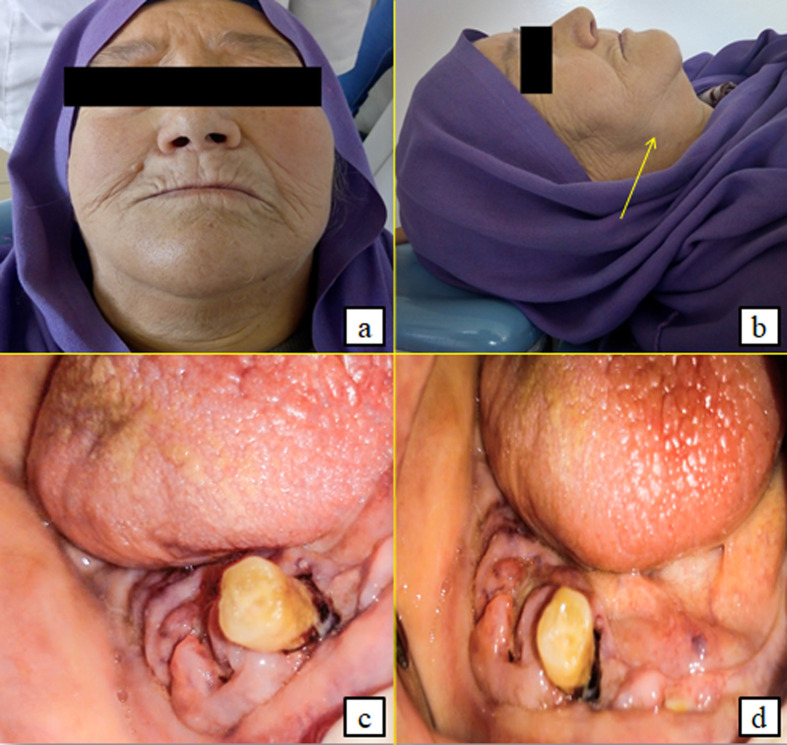
extraoral presence of a palpable right submandibular lymphadenopathy (A,B) and intraoral ulcerated and budding right gingivo-mandibular lesion extending throughout the mandibular crest and evolving for 1 month (C,D)

**Figure 2:**
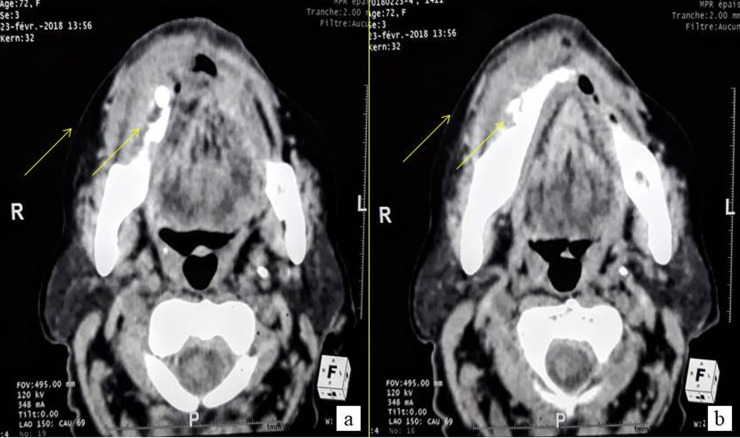
craniofacial CT-scan: axial sections, narrow window

**Figure 3:**
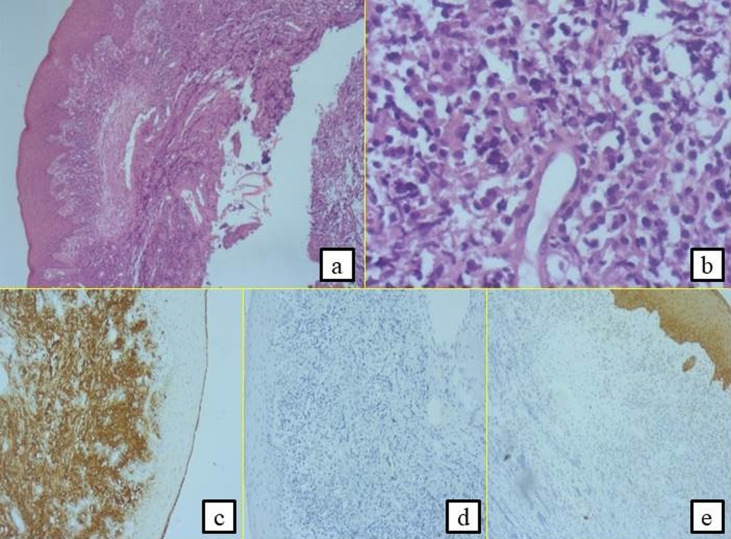
histological and immunohistochemical examinations leading to the diagnosis of large B-cell lymphoma; presence of large tumour cells having clear and abundant cytoplasm with nuclei showing several marked atypias (A,B). The mitoses and the apoptotic bodies were quite numerous; the tumour was the site of important necrotic changes, the tumour cells strongly expressed CD20 (C); they did not express CD3 (D) and CK (E)

**First case:** a 72-year-old female patient with a history of insulin-dependent diabetes mellitus, hypertension, gout and ischemic heart disease who had undergone coronary angioplasty was referred to the oral medicine and oral surgery department at SAHLOUL Hospital (Sousse, Tunisia) because of pain and continuous discomfort localized in the right mandibular region. The extra oral clinical examination revealed the presence of one palpable right submandibular lymph node greater than 1 cm and having a firm consistency (Figure 1, A,B). The intraoral examination revealed insufficient hygiene with the presence of a gingival bleeding lesion having locally-ulcerated raised edges in relation to the second premolar (45) with a class 3 mobility grade. This lesion extended throughout the right mandibular crest and had been evolving for 1 month, according to the patient (Figure 1, C,D). The neurological examination revealed a unilateral labial-chin hypoaesthesia suggestive of malignancy. The craniofacial CT scan examination revealed an infiltration of the right lateral mandibular soft tissues associated with a poorly-limited osteolytic lesion affecting the mandibular crest in relation to the floating tooth 45 (Figure 2). It was associated with bilateral multiple lymph nodes (submandibular, supraclavicular and jugulo-carotid), the largest of which measured 20 mm. A biopsy was performed. The histological and immunohistochemical examinations led to the conclusion that the tumour is a large B-cell lymphoma of the mandibular gingiva. Histologically, there were large tumour cells having clear and abundant cytoplasm with nuclei showing several marked atypias. The mitoses and the apoptotic bodies were quite numerous. The tumour was the site of important necrotic changes. In immunohistochemistry, the tumour cells strongly and diffusely expressed CD45 and CD20. They did not express CD3 and CK (Figure 3). The patient was referred to the onco-haematology department where a chemotherapy-based treatment was initiated. She showed a clear improvement in her condition after the first cycles.

**Figure 4:**
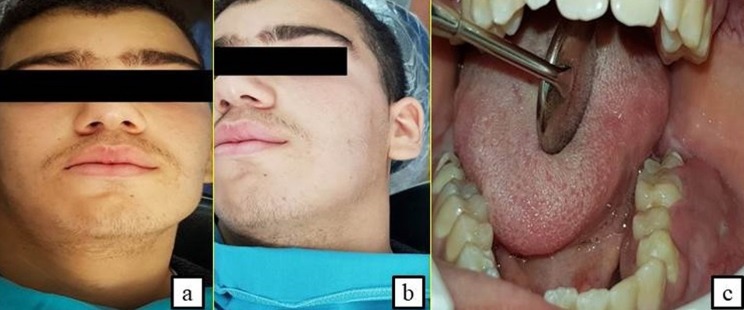
extraoral slight swelling on the left cheek + left palpable cervical lymphadenopathy with sensation of hypoaesthesia along the path of the left inferior alveolar nerve (A,B) and intraoral extended gingival mass ranging from the 34 to 38 with terminal dental mobility (C)

**Figure 5:**
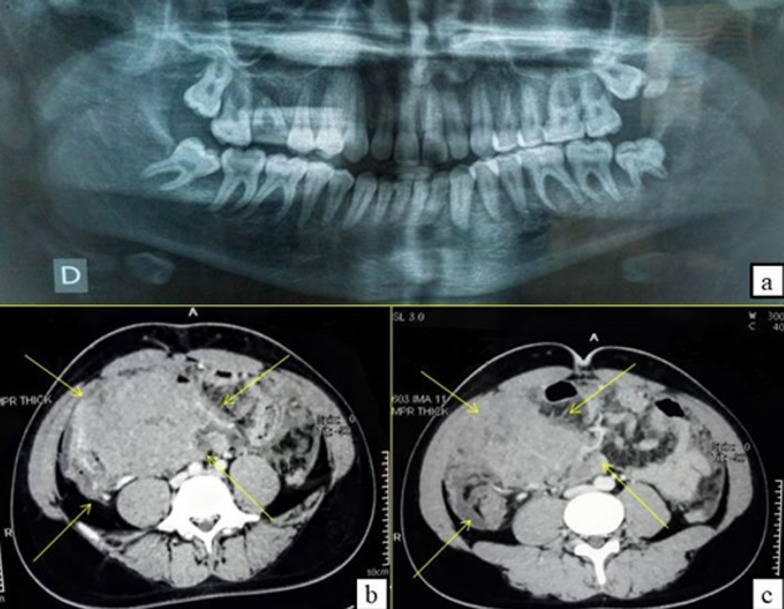
panoramic X-ray invasion of the teeth furcation and bone lysis in the left molar region (B) and abdominal CT-scan (B,C): axial sections, narrow window: tumour mass of the ileocecal junction associated with peritoneal carcinomatosis, presence of multiple mediastinal lymphadenopathies

**Figure 6:**
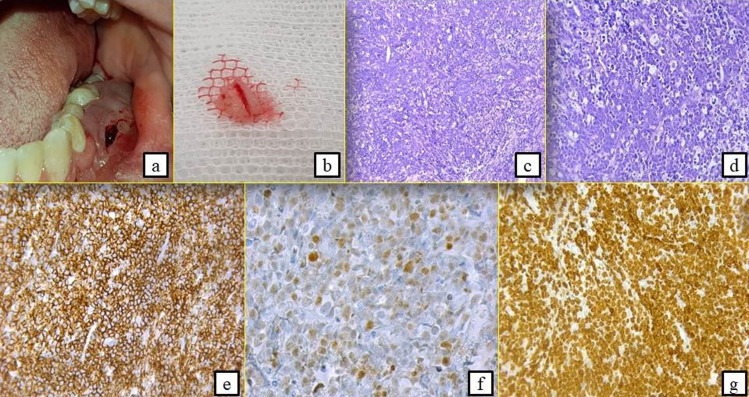
oral biopsy: left mandibular attached gingiva in the swelling next to the premolars (A,B); histological and immunohistochemical examinations leading to the diagnosis of Burkitt´s lymphoma (C,D,E,F,G); presence of a lymphomatous proliferation of diffuse architecture consisting of small to medium lymphocytes, with basophilic cytoplasm with too many mitoses (A,B); the lymphocytes were of phenotype B expressing markers CD20 (+) (E) and heterogeneous Bcl6 (F); the tumour proliferation index Ki67 was 100% (G)

**Second case:** a 16-year-old male patient with acute articular rheumatism and a history of erysipelas at the age of 7 years was hospitalized in the General Surgery Department because of a deterioration of his general condition. Originally, the patient suffered from epigastralgia, vomiting and an influenza state for which he had a symptomatic treatment. This was aggravated by a progressive onset of pain in the right iliac fossa with diarrhea-like transit disorders. He was referred to our department to manage a left mandibular swelling with a suspected abscess of dental origin. The extraoral clinical examination revealed the presence of palpable left cervical lymphadenopathy with normal mouth opening (Figure 4, A,B). The intraoral examination showed an extensive gingival mass ranging from teeth 34 to 37 with terminal dental mobilities evolving for 3 weeks (Figure 4C). On the neurological examination, the patient described a sensation of hypoaesthesia along the path of the left inferior alveolar nerve. The radiological examination revealed the presence of a bone destruction involving teeth furcation in the left molar region (Figure 5A). The abdominal CT scan evoked a tumoural mass of the ileocecal junction associated with a peritoneal carcinomatosis as well as multiple mediastinal lymph nodes, the largest of which measured 31*21mm (Figure 5,B,C). The biological analyzes showed a significant increase in CRP values at 117 and in LDH at 789 (Table 1), indicating the existence of a biological inflammatory syndrome and tissue damage in the body. HIV serology was negative. In this context, an association between the oral lesion and the abdominal tumour was suspected, suggesting a cancer of lymphomatous origin. A biopsy of the oral lesion was performed (Figure 6, A,B) and the patient was surgically operated for his abdominal tumour in the general surgery department. The results of the anatomopathological examinations were in agreement with our suspicion. It was a Burkitt lymphoma with primary abdominal and secondary oral localisations. Histologically, there was a lymphomatous proliferation of diffuse architecture consisting of small to medium lymphocytes, basophilic cytoplasm with too many mitoses. In the immunohistochemical study, the lymphocytes were of phenotype B expressing markers CD20 (+), CD10 (+) and heterogeneous Bcl6. The tumour proliferation index Ki67 was 100% (Figure 6 C,D,E,F,G). The postoperative follow-up was straightforward and the patient was referred to the onco-haematology department where a chemotherapy treatment was initiated. Unfortunately, the patient died one month later.

**Table 1:** the CRP and LDH values

	Normal value	Patient value
CRP (mg/l)	<8	117
LDH (UI/l)	140-271	789

## Discussion

Accounting for about 12% of all malignant tumours of the head and neck region, lymphomas are actually the third most frequent cancer after squamous cell carcinoma (46%) and thyroid carcinoma (33%). Thus, they should always be considered in cases of suspicious cervical or oral masses [1]. Diagnosis of NHL can be raised in front of several clinical signs like persistent superficial painless lymph nodes at any location, hepatomegaly or splenomegaly at an unusual context and general signs such as weight loss, fever and profuse nocturnal sweats. Biological analyses may reveal an unexplained inflammatory syndrome, sometimes an abnormality of the hemogram (cytopenia, hyper lymphocytosis, etc.) and an increase in LDH rate [7]. Most of these clinical (impaired general condition, persistent lymph nodes...) and biological signs (high increase of CRP and LDH) were reported in our patients. The extranodal manifestations occur in 40% of the cases at various sites (oral, gastric, cutaneous, cerebral...) [7]. Lymphomas of the oral cavity constitute 2% of extranodal lymphomas. The palate, maxilla and gingiva are the most common locations of these diseases. Involvement has also been reported in the floor of mouth and tongue [4]. The soft tissues are usually more involved than the jaws [8]. However, we found intraosseous lesions in the reported cases. The mandibular locations of non-Hodgkin's lymphomas are rare and represent only 0.6% of the reported cases [3], which makes our observations original. However, these circumstances are not very specific [3] and they can mislead the clinician, make the diagnosis difficult and delay the treatment. It is important to note that only a deep and good quality tumour biopsy can provide a diagnosis of certainty.

The standard imaging assessment includes a thoracic, abdominal and pelvic computed tomography, with measurement of the lesions that will serve as the initial reference for the treatment response assessment. A PET scanner FDG is also necessary to identify the degree of tumoural extension and to determine the Ann Arbor stage [7]. The two cases reported in this paper represent the two highly aggressive known NHLs according to the WHO classification. Diffuse large B-cell lymphoma (DLBCL) is the most common type of non-Hodgkin lymphoma originating from the germinal center and representing approximately 60% of cases each year [3]. It most often presents as a rapidly enlarging, single nodal or extranodal mass [4]. Among the different lymphomas, it also shows the highest prevalence in the oral cavity. The disease is aggressive and it affects patients over 60 years of age as it is reported in the first case, but this tumour can also be observed in children. The disease evolves spontaneously towards a gradual extension and worsening leading to a fatal outcome in a few months. This therefore implies the importance of treatment initiation from the initial management. Morphologically, Diffuse Large B-Cell Lymphoma (DLBCL) is characterized by a diffuse infiltration of medium to large cells with large nucleoli and abundant cytoplasm which disrupts and effaces the underlying architecture of the involved tissue. The cells typically express pan-B cell antigens, including CD19, CD20, CD22, CD79a and CD45. The majority of the cells also express surface immunoglobulin. Approximately 14% of the cases express CD30, which can portend a favourable prognosis [9]. In our case the cells strongly expressed CD20 and CD45 antigens. They did not express CD3 which is specific to NHL of T-cell subtype or cytokeratin (CK) which excluded the diagnosis of a squamous cell carcinoma.

As for Burkitt's lymphoma, it is a highly aggressive B-cell non-Hodgkin´s lymphoma that accounts for more than 80% of NHL in the children [10]. Actually, three major subtypes of this malignant tumour exist: Endemic, Sporadic, and Immunodeficiency-associated [11]. The endemic form (African variety) was first recognized in Uganda in the 1950's by a pathologist called Dennis Burkitt as a tumour affecting the jaw and abdomen in children. This form is characterized by a constant association with Epstein Barr virus. Sporadic BL is considered non-endemic, occurring in scattered parts of the world where EBV infection is not prevalent such as the United States (US) and Europe. Immunodeficiency-associated BL affects patients infected with human immunodeficiency virus (HIV) as well as organ transplant recipients. Disease presentation differs between the different types [12]. Histologically, BL features are similar in the three clinical variants. They consist of an infiltrate of rather monomorphic intermediate-sized lymphocytes with scant amphophilic to basophilic cytoplasm and fine nuclear chromatin. There tends to be a “starry-sky” pattern with accompanying tingible body macrophages similar to what was seen in our case. BL expresses pan B-cell antigens CD19, CD20, CD22, CD79a, CD10, CD38 and BCL6. It is negative for BCL2 [13]. The Ki-67 proliferation index approaches 100% which was found in our case. The following immunohistochemical characteristics were present in our case (expression of CD20 and CD10, heterogeneous BCL6, Negative BCL2 and Ki-67=100%).

Since our patient was not clinically and biologically diagnosed with a viral infection (EBV or HIV) and histologically there was not a positive staining for EBV, we can consider that we are in front of the sporadic form of BL with a bulky abdominal mass, multiple lymphadenopathy and an extranodal involvement of the oral cavity. The disease staging and the evaluation of the prognostic factors represent a fundamental step in the patient´s management, essential for treatment decisions. The reference treatment is medical consisting in the introduction of chemo-immunotherapy (CI) with R-CHOP because of the high chemosensitivity of the tumour: It combines 6 to 8 cycles of multidrug therapy according to the CHOP protocol containing Cyclophosphamide, Adriamycin, Vincristine and Prednisone with a monoclonal anti-CD20 antibody that is Rituximab [7]. The cycles are spaced 2 to 4 weeks apart. Even when cure is not possible, this treatment is capable of controlling the disease for many years. Further chemotherapy can often be given if the disease relapses after the initial treatment. Prognosis depends on the degree of the initial extension and the speed of the treatment initiation. Since 1993, clinicians have used the International Prognostic Index (IPI) to characterise prognosis in aggressive NHL based on five clinical factors: age, stage, number of extranodal sites, performance status according to the scale of the Eastern Cooperative Oncology Group (ECOG) and LDH (Table 2) [14]. As for our patients, prognosis was moderately favourable for the first one but very poor for the second one because the tumour was diagnosed in a very advanced stage.

**Table 2:** the clinical prognosis factors for each patient

	Sth	Unfavourabe if	Patient 1	Patient 2
International Prognosis Index (IPI)	Age	> 60 years	Yes	No
	Ann Arbor stage	III or IV	II	IV
	Extranodal sites	>2	No	Yes
	Performance Status	>2	No	Yes
	LDH	>Normal Value	Not determined	Yes

The complete remission rate of Non-Hodgkin´s lymphomas after chemotherapy is 60 to 80% per year [3]. The patient´s follow-up is indispensable because of the disease recurrence risk which must not be neglected [15]. It is provided by the referring team working closely with the treating doctor. Recurrence can be nodal or extra-nodal; whatever the initially affected site is. Any unusual and persistent clinical element must evoke a relapse. The patient must be informed of the suspicious clinical signs suggestive of the disease evolution in order to seek treatment as quickly as possible. Most often, oral involvement is an early manifestation of the development of nodal non-Hodgkin lymphoma [14]. In either case, dental pain associated with swelling refractory to any treatment is the first evocative sign. In case of diagnostic uncertainty, a biopsy should be performed. The presence of malignancy signs such as rapid and sudden evolution, associated lymph nodes, labial-chin hypoaesthesia and features outside the usual classic signs of a squamous cell carcinoma such as wartiness, ulceration and induration of lesions may evoke lymphoma. The reported observations illustrate the importance of the oral surgeon´s role in early diagnosis because a persistent oral lesion is often the only symptom of this challenging disease. Its management must be multidisciplinary and may involve various medical specialties. Errors often result from an incomplete approach. A delay in diagnosis is detrimental to the patient when he is not quickly managed.

## Conclusion

Mandibular non-Hodgkin´s lymphomas are rare but they should be included in the differential diagnosis of other jaw lesions. This paper reported two original entities of the highly aggressive grade of these diseases. Diagnosis is always challenging due to the lack of specific symptoms which may lead to misdiagnosis and therefore delay the initiation of the appropriate therapy. Biopsy must always be performed in case of suspicious findings following meticulous clinical examination. As oral surgeons, we have an important role in the early diagnosis of these malignancies and in the patient´s referral to specialized care in order to get the appropriate treatment.
